# A Rare Case of Paediatric Neck Swelling: Cervical Sympathetic Chain Schwannoma

**DOI:** 10.1155/2013/712365

**Published:** 2013-04-30

**Authors:** E. Keane, E. C. Francis, Sri Paran Thambipillai

**Affiliations:** Department of Surgery, Our Lady's Hospital for Sick Children, Crumlin, Dublin 8, Ireland

## Abstract

Schwannomas are indolent benign lesions arising from schwann cells in the nerve. They are especially rare in the paediatric population. We report an interesting case of a swelling in the upper neck, which highlights an atypical presentation of this tumour, as well as the complex details of its clinical, radiological, and surgical management.

## 1. Introduction

Schwannomas are indolent benign lesions arising from schwann cells in the nerve. Their earliest description was at the start of the twentieth century, and they were referred to as neurinomas [1]. They are a rare occurrence, and only up to twenty-five percent are reported in the head and neck region in adults [2]. They are especially rare in the paediatric population. They have a predilection for the origin of vagus nerve in the neck but may be found arising from any other nerve with a schwann sheath. When arising from the cervical sympathetic chain, they are usually found in the superior and middle portions [3]. Schwannomas have a male preponderance and may present at any age. However, they frequently occur in middle age with a wide range between thirty to seventy years reported. We report a case manifesting as a swelling in the upper neck, which confirms the typical presentation of the tumour, and detail its clinical, radiological, and surgical management.

## 2. Case Report

**Figure 1:**
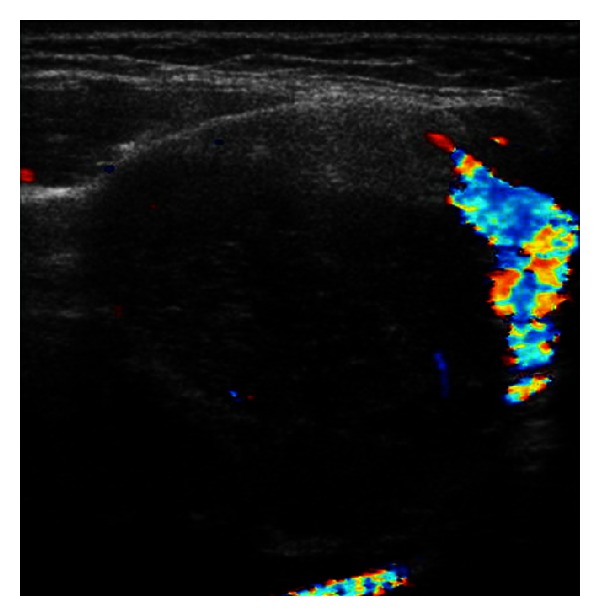
Ultrasound right neck mass.

**Figure 2:**
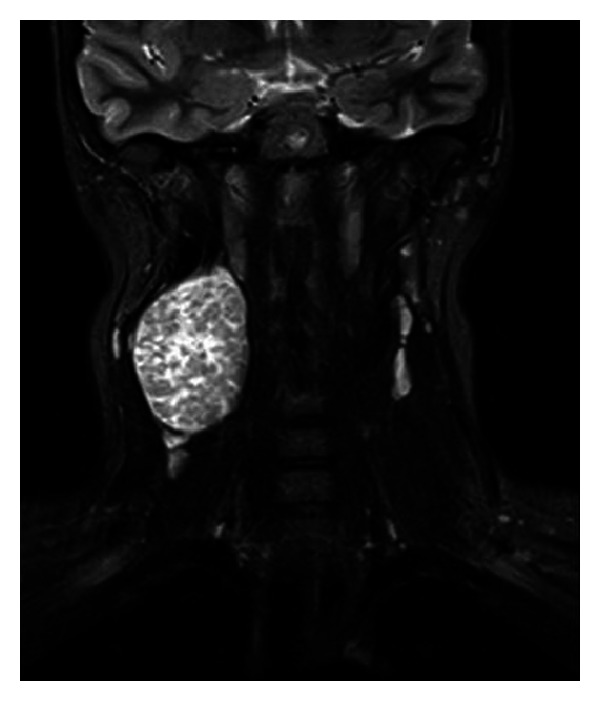
MRI head and neck showing a 3.9 × 3.8 × 6.1 cm lesion with a heterogeneous enhancement pattern and cervical lymph nodes within normal limits.

A fourteen-year-old girl presented with a one-year history of an asymptomatic slowly enlarging mass on the right side of her neck. There was no history of hoarseness, nasal regurgitation or associated pain, fever, or trauma. Her medical history was significant for recurrent tonsillitis in early childhood, but the mass was found to be unresponsive to over three courses of oral antibiotics before being referred to our specialist centre for evaluation. On examination, she had a large six-by-four centimetre immobile solitary right-sided neck mass deep to the right sternocleidomastoid muscle with a smooth regular border. There was no associated palpable cervical lymphadenopathy. The patient's oropharynx revealed no displacement of the peritonsillar structures, and a further physical examination was non-contributory. Routine blood tests including LDH were normal. Ultrasound showed a solid, well-circumscribed homogeneous mass arising from within the fascial planes with low level Doppler venous flow. The mass was displacing the great vessels to the right (Figure 1). She went on to have an MRI (Figure 2).

**Figure 3:**
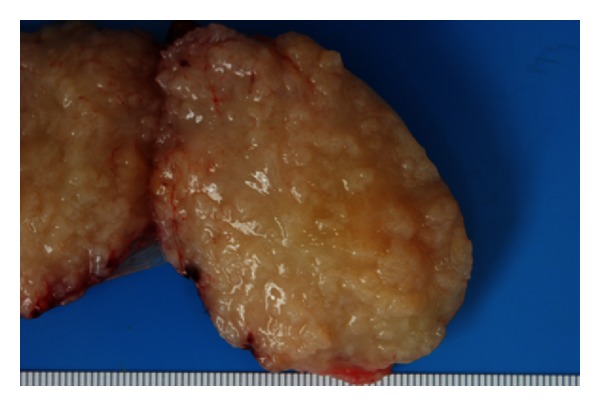
Gross specimen of the mass longitudinally sectioned. The capsule can be clearly seen with the pale tissue and irregular nodular cut surface.

This patient was discussed at our joint radiology/surgical MDT, and a consensus was made to proceed with surgical resection of the mass. During surgery, there were two lymph nodes above the right internal jugular vein identified which were excised for histopathology. These were later confirmed to be reactive hyperplasia. The mass was identified posterior to the right internal jugular vein and mobilised with blunt and bipolar dissection medially off the carotid artery (Figure 3), and laterally off the scalene muscles. It was arising from the cervical sympathetic chain. The nerve was divided to facilitate complete resection of the mass; however, the remainder of procedure was unremarkable. 

**Figure 4:**
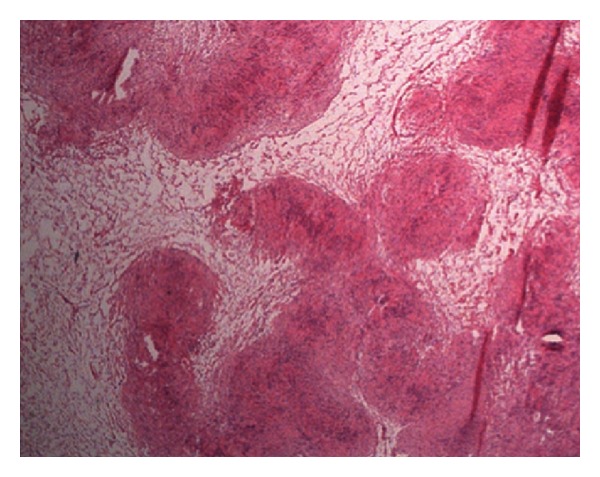
Microscopic appearance of spindle cell tumour.

Postoperatively the patient developed anisocoria and ptosis of her right eye. A diagnosis of Horner's syndrome was made, the description of which forms a vital part of the informed consent process prior to surgical resection. The final histology report confirmed a palisaded spindle cell lesion consistent with a nerve sheath tumour (Figure 4).

## 3. Discussion

Schwannomas are solitary benign indolent tumors that commonly occur in individuals with neurofibromatosis. Schwann cells are glial cells that myelinate the axons of nerve cells. When schwann cells proliferate out of control in a capsule, it is called a schwannoma. Though benign, they can sporadically undergo malignant transformation. They can become debilitating when the growing tumor compresses the nerve causing chronic severe pain [4]; however, they typically present as painless, asymptomatic neck mass as in our case. This is due to the fact that the cervical sympathetic chain runs in a relatively loose fascial compartment and compression is rare.

The head and neck region is the most common site of origin of schwannomas, but they can develop from any peripheral, cranial, or any autonomic nerve that has a schwann sheath. In the head and neck, they arise medially from glossopharyngeal, vagus, accessory, or hypoglossal nerves or, as is this case, from the sympathetic chain. Laterally they may arise from the brachial or cervical plexus [5]. Schwannomas that originate from the cervical sympathetic chain are rare with less than fifty cases reported to date in the literature [6].

The cervical sympathetic ganglia are part of the sympathetic chain in the neck region and run longitudinally over the longus capitis and longus colli muscles, as far as the pre-vertebral fascia. They are comprised of three ganglia—the superior, middle, and the inferior ganglia. The superior ganglion is largest and lies at the level C2-4, the middle ganglion lies at the level of C6, while the inferior ganglion is variable in position. It can fuse with the first thoracic ganglion to form a stellate ganglion. In most cases reported in the literature, the SCSC arises from the superior or middle part of the sympathetic chain [4, 7].

Horner's syndrome is a common postoperative complication due to the close association between the nerve and the tumour, which makes surgical separation almost impossible in the majority of cases. There has been some evidence to support intracapsular enucleation in order to minimise the risk of nerve of origin palsies following resection [8]. While conservative surgical excision is the therapeutic option of choice in benign lesions, there is no established consensus on the optimal treatment of malignant schwannomas. Most authors, however, agree that intervention is dependent on tumor size and histologic grade [9, 10].

In their case series of eight tumours, Colreavey and colleagues noted six benign and two malignant schwannomas. One of their eight cases (twelve percent) presented with a horner's syndrome and that malignant schwannomas were rare and often associated with familial disorders such as neurofibromatosis [11]. The differential diagnoses of these lesions are broad. They include chemodectomas, parotid tumours, salivary gland tumours, lymphoma, branchial cleft cyst, vagal schwannoma, distant metastases, neurofibroma, aneurysms of the internal carotid artery and patients should undergo salient diagnostic examinations to rule these out [12].

In summary, this particular case is important as it is the first reported case of a schwannoma of the cervical sympathetic chain in Ireland in the paediatric population. Secondly, it reiterates that the management does not differ between adults and children who present with these tumours and that complete surgical excision is the mainstay of treatment for both diagnostic and therapeutic purposes. The prognosis of these lesions is excellent, and recurrence is rare.
